# The Tension and Puncture Properties of HDPE Geomembrane under the Corrosion of Leachate

**DOI:** 10.3390/ma6094109

**Published:** 2013-09-17

**Authors:** Qiang Xue, Qian Zhang, Zhen-Ze Li, Kai Xiao

**Affiliations:** State Key Laboratory of Geomechanics and Geotechnical Engineering, Institute of Rock and Soil Mechanics, Chinese Academy of Sciences, Wuhan 430071, China; E-Mails: zq_cersm@163.com (Q.Z.); xuerundan@163.com (Z.-Z.L.); xk_cersm@163.com (K.X.)

**Keywords:** landfill, corrosion of leachate, HDPE geomembrane, tension properties, puncture properties

## Abstract

To investigate the gradual failure of high-density polyethylene (HDPE) geomembrane as a result of long-term corrosion, four dynamic corrosion tests were conducted at different temperatures and durations. By combining tension and puncture tests, we systematically studied the variation law of tension and puncture properties of the HDPE geomembrane under different corrosion conditions. Results showed that tension and puncture failure of the HDPE geomembrane was progressive, and tensile strength in the longitudinal grain direction was evidently better than that in the transverse direction. Punctures appeared shortly after puncture force reached the puncture strength. The tensile strength of geomembrane was in inversely proportional to the corrosion time, and the impact of corrosion was more obvious in the longitudinal direction than transverse direction. As corrosion time increased, puncture strength decreased and corresponding deformation increased. As with corrosion time, the increase of corrosion temperature induced the decrease of geomembrane tensile strength. Tensile and puncture strength were extremely sensitive to temperature. Overall, residual strength had a negative correlation with corrosion time or temperature. Elongation variation increased initially and then decreased with the increase in temperature. However, it did not show significant law with corrosion time. The reduction in puncture strength and the increase in puncture deformation had positive correlations with corrosion time or temperature. The geomembrane softened under corrosion condition. The conclusion may be applicable to the proper designing of the HDPE geomembrane in landfill barrier system.

## 1. Introduction

The high-density polyethylene (HDPE) geomembrane is a major part of the barrier system in a landfill that helps to inhibit the diffusion of leachate from polluting groundwater. The long-term reliability of the HDPE geomembrane is the key to guaranteeing the ecological security of a landfill. The permeability coefficient of an intact HDPE geomembrane is generally less than 1.0 × 10^−14^ m/s, which is typically considered impervious and exhibits good anti-seepage performance. In addition, the HDPE geomembrane has stable chemical properties, corrosion resistance, and relatively mature experience in project implementation [1]. However, large deformations, puncture holes, and tears occur under complicated pressures generated by the landfill refuse dump because the HDPE geomembrane is prone to uneven settlement and punctures. The waste body itself with a complex composition of gravel, glass, and other hard objects can easily break the membrane. The mechanical properties of materials, such as the tensile and puncture strength, determine the sealing effect of the HDPE geomembrane in landfill application. Although the geomembrane has good chemical stability and corrosion resistance, tensile and puncture strength of the geomembrane gradually decreases, which affects the barrier performance as a result of long-term exposure to leachate and heat released by bio-reaction inside the landfill waste. Therefore, it is imperative to develop tests methods on the tension and puncture properties of HDPE geomembrane under the corrosion of leachate at different durations and temperatures.

Studies on the mechanical deformation characteristics and other issues related to HDPE geomembranes in engineering applications have been conducted through experiments, theories, and numerical simulations. However, these studies mainly focused on the interface properties of geomembrane and other earthwork synthetic materials [2,3,4,5,6,7,8,9,10,11,12,13,14,15], of which very few was involved with the tensile and puncture properties of geomembranes [16,17,18,19]. Particularly rare tests were developed to determine the effect of landfill temperatures on tension and puncture properties [20,21,22]. In this study, a stock solution of leachate was obtained from the Wuhan Chenjiachong landfill in winter. The effects of leachate on the mechanical properties of HDPE geomembrane were examined based on the typical temperature changes inside the landfill. The varying behavior of the tensile and puncture properties of the HDPE geomembrane at different corrosion durations and temperatures were analyzed by a dynamic corrosion instrument CERSM-X20 that was developed in our group and has been applied to study polluted mass (*i.e*., rock/earthwork synthetic materials). The conclusions reached in this study can provide critical date support for the quantitative evaluation of HDPE geomembrane as well as the designing and optimization of landfills.

## 2. Materials and Methods

### 2.1. Experimental Materials

#### 2.1.1. HDFE Geomembrane

The HDFE geomembrane used in this study was obtained from High-Energy Era Environment Technology Co. Ltd. (Beijing, China) and was made of high-density polyethylene resin, which was extruded to extrusion sheet. No obvious holes or cracks were found on the surface of geomembrane, satisfying the industrial standards for application in landfill barriers. The fundamental parameters of the HDPE geomembrane are presented in Table 1.

**Table 1:** Parameters of HDPE geomembrane.

Parameters	Thickness	Density	Tensile strength (crossbar)	Elongation at break (crossbar)
HDPE geomembrane	1 mm	0.95 g/cm^3^	≥25 MPa	≥550%
**Parameters**	**Tangential breaking strength (crossbar)**	**Permeability coefficient of water vapor**	**Oxidation induction time in 200 °C**	–
HDPE geomembrane	≥110 N/mm	<1.0 × 10^−16^ m/s	≥20 min	–

#### 2.1.2. Landfill Leachate

The Stock solution of landfill leachate was obtained from the Wuhan Chenjiachong landfill in winter. The chemical components were analyzed by relevant instruments and are shown in Table 2.

**Table 2:** Chemical components contents of landfill leachate.

Chemical components	pH	COD	BOD	TOC	Cl^−^	Na^+^	NH_4_–N	Pb^2+^	Cd^2+^
Contents	8.56	21562	871.2	9032	2708	852	746.8	0.17	0.107
Chemical components	Cu	Fe	Zn	Phenol	Ca^2+^	K^+^	Mg^2+^	Hardness(CaCO_3_)	SO_4_^2−^
Contents	0.11	7.26	0.48	0.1117	2072	794	1537	3401	385

### 2.2. Test Methods

The geomembrane specimens for the tensile strength test were cut into strips at 50 mm × 200 mm in size, and the specimens for puncture strength were circular with a diameter of 100 mm. Under normal condition, the corrosion rate was extremely low. To shorten the test time, a dynamic corrosion method was applied to accelerate the corrosion. A novel dynamic corrosion instrument centrifugally stirred the specimens. The temperature and pressure in corrosion tests could be controlled through temperature control system and pressure system. The prepared specimens were placed into the CERSM-X20 reactor at first, into which the landfill leachate was added. The centrifugal rotation speed was 300 r/min. And the air pressure in the reactor was kept at 20 MPa. Three corrosion temperatures of 20, 50 and 80 °C, as well as three corrosion times of 5, 10 and 15 days, were carried out separately. Specimens were taken out of the leachate when the corrosion time was reached, and then the Tensile and puncture strengths of corroded specimens were tested immediately by using the traditional testing machine equipped with load cell, cross-head monitor and self-aligning wedge grips. Because the time needed for the tension and puncture tests was much less than the corrosion time, the impact of that period of time without corrosion was negligible. Tension and puncture tests were conducted at room temperature (22 °C).

Two parallel lines perpendicular to the stretching direction were drawn 100 mm apart on the tension specimens beforehand. The two lines were as close to the upper and lower edges of the clamps as possible to ensure the gauge length of test specimen was 100 mm. The stretch rate was 20 mm/min. The running speed of machine for puncture tests was 300 mm/min, and the center line was ensured to be on the axis for puncture specimens. Other factors involved in the test were consistent.

The effects of corrosion temperatures and durations on the tension and puncture properties of the HDPE geomembrane were investigated in this study. Eighteen sets of specimens were prepared for the tensile tests, and nine sets of specimens were prepared for puncture tests under different conditions. The test conditions are presented in Table 3.

**Table 3:** Working conditions of HDPE geomembrane for tests.

Test conditions		Tensile test at longitudinal grain direction, *z*			Tensile test at cross grain direction, *h*			Puncture test		
		Corrosion time, *t* (days)			Corrosion time, *t* (days)			Corrosion time, *t* (days)		
		5	10	15	5	10	15	5	10	15
Temperature, *T*	20 °C	*z*-20-5	*z*-20-10	*z*-20-15	*h*-20-5	*h*-20-10	*h*-20-15	20-5	20-10	20-15
	50 °C	*z*-50-5	*z*-50-10	*z*-50-15	*h*-50-5	*h*-50-10	*h*-50-15	50-5	50-10	50-15
	80 °C	*z*-80-5	*z*-80-10	*z*-80-15	*h*-80-5	*h*-80-10	*h*-80-15	80-5	80-10	80-15

The tension and puncture tests on the intact specimens were conducted on the same conditions and were considered as the control test. The test data were compared with the data from corroded specimens to evaluate the influence of corrosion on the tension and puncture strength of specimens.

Tension and puncture tests were conducted in accordance with SL/T 235-1999 [23] and GB/T 19978-2005 [24].

## 3. Results and Analysis

### 3.1. The Tension and Puncture Properties of Intact Geomembrane Specimens

The failure mode of geomembrane was progressive, and necking occurred randomly in the middle of the geomembrane specimens during the stretch process to form a damaged extending zone. The damaged extending zone developed at both ends with the increase in tension, and the damage zone narrowed at the middle and widened at both ends upon termination of the test. The HDPE geomembrane exhibited high ductility and could be extended by two to three times the length of the original without fracturing.

**Figure 1:**
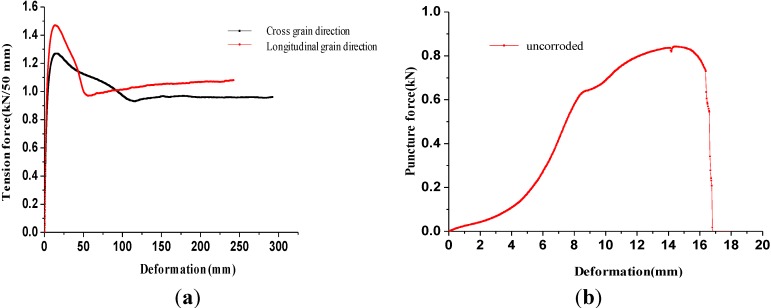
The tension and puncture properties of uncorroded geomembrane specimens.

Figure 1a illustrates the tension force-deformation curves of the intact geomembrane specimens. The tension force-deformation curves of the geomembrane specimens quickly reached the peak strength, then gradually declined to the lowest strength, and finally increased to the horizontal trend regardless of whether the tension was in the longitudinal or the transverse direction. The final strength remained relatively constant, which corresponded to the residual strength. The law exhibited in the tension force-deformation curves was in agreement with the deformation features and failure modes. Necking did not occur before the geomembrane specimens reached the tensile strength. The strength of specimens began to decline when necking occurred, and the damaged extending zone began to develop at two ends when strength decreased to the lowest. Strength then rose at small amplitude until the specimen strength reached the residual strength. Finally, strength remained relatively constant. The HDPE geomembrane consisted of numerous crisscrossed high polyethylene chains, the directional trend of which gradually improved under the tension force. The original disordered chains tended to have a more parallel distribution, which caused geomembrane specimens to undergo progressive failure. The improvement in directional trend slightly increased the strength of the geomembrane specimens and was the main reason for the increase of the strength to the residual strength after it dropped to the lowest level. The strengths of the uncorroded geomembrane specimens in the longitudinal and transverse direction were respectively 1.47 and 1.27 kN/50 mm. Residual strengths were determined at 1.08 and 0.96 kN/50 mm. The elongation was at 13.2% and 14.8%. The strength in the longitudinal grain direction was obviously larger than the strength in the transverse direction.

In the puncture test, the centers of the geomembrane specimen moved down with the cone head, but the specimens were punctured right away. Instead, a circular depression with the specimen center as the middle of the circle was formed because of the good ductility of geomembrane. Puncture failure was also progressive, and the specimens could be punctured through only when the deformation increased by a certain degree.

Figure 1b indicates the puncture force-deformation curve of the uncorroded geomembrane. As the figure shows, the puncture force of the geomembrane had a short, slow decline process after reaching the puncture strength and then was rapidly reduced to zero as the geomembrane specimen was punctured through. The change of the puncture force-deformation curve was the same as the deformation failure modes. As a result of the progressive failure mode of the geomembrane, the puncture did not occurred when puncture force increased to the puncture strength. Instead, it occurred after the puncture force slightly decreased. The puncture strength of the uncorroded geomembrane was 0.843 kN, and the puncture deformation was 14.5 mm.

### 3.2. Impact of Corrosion Time on the Tensile Strength Properties of Geomembrane Specimens

**Figure 2:**
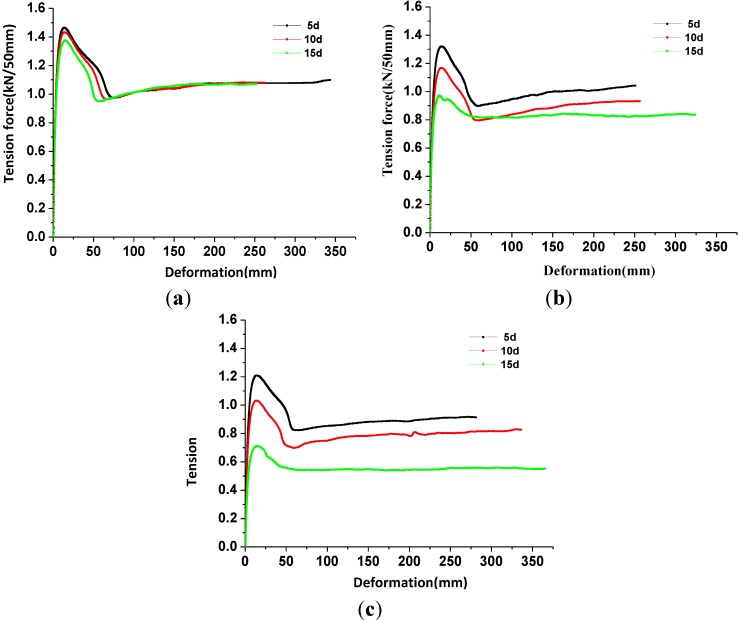
The tension force-deformation curves of geomembrane specimens in the longitudinal grain direction. (**a**) 20 °C; (**b**) 50 °C and (**c**) 80 °C.

**Figure 3:**
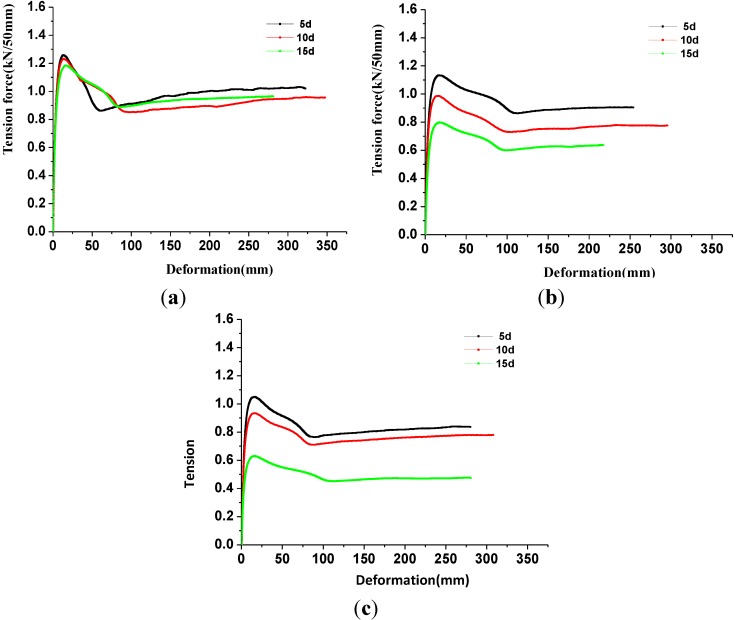
The tension force-deformation curves of geomembrane specimens in the cross grain direction. (**a**) 20 °C; (**b**) 50 °C and (**c**) 80 °C.

Figure 2 and Figure 3 show the tension force-deformation curves of geomembrane specimens under different corrosion conditions in the longitudinal and cross grain direction, respectively.

Figure 2a shows that the corresponding tensile strengths of the geomembrane specimens in the longitudinal grain direction were 1.466, 1.434 and 1.375 kN/50 mm, respectively, after being corroded with leachate for 5, 10, and 15 days at 20 °C. In Figure 3a, the corresponding tensile strengths of the geomembrane specimens in the cross grain direction were 1.258, 1.231 and 1.182 kN/50 mm, separately. The tensile strengths in the longitudinal grain direction decreased by 0.27%, 2.45% and 6.46% compared with the uncorroded geomembrane specimens. The tensile strengths in the cross grain direction decreased by 0.94%, 3.07% and 6.93%. All of the tensile strengths were proven to decrease with the increase of corrosion time regardless of whether the geomembrane specimens were in the longitudinal or the cross grain direction. The changes in the cross grain direction were greater than those in the longitudinal grain direction under the 20 °C condition. However, the ratio of tensile strength attenuation was negligible particularly for the specimens that were corroded for 5 days mainly because of the oxidative induction effects of the antioxidant in the geomembrane. The consumption of antioxidant in the geomembrane was measured by oxidative induction time [25]. The attenuation of antioxidant could make the crystallization of geomembrane much easier, which would decrease the tensile strength under dynamic corrosion condition. Rowe *et al.* [26] reported that the higher the temperature of the geomembrane, the faster the oxidative induction time decreased. Oxidation induction time decreased slowly at 20 °C, which caused changes in the tensile strength that were not obvious. As Figure 2b shows, the corresponding tensile strengths of the geomembrane specimens in the longitudinal grain direction are 1.321, 1.168 and 0.973 kN/50 mm, respectively, after being corroded with leachate for 5, 10 and 15 days under the 50 °C condition. As Figure 3b indicates, the corresponding tensile strengths of the geomembrane specimens in the cross grain direction are 1.133, 0.986 and 0.797 kN/50 mm. The tensile strengths in the longitudinal grain direction decreased by 10.14%, 20.54% and 33.81% compared with the 20 °C condition. The tensile strengths in the cross grain direction decreased by 10.79%, 22.36% and 37.24%, and the attenuation of the oxidative induction time accelerated. The tensile strength also decreased with corrosion time, and the changes in the cross grain direction were greater than in the longitudinal grain direction. The aforementioned law was also met under the 80 °C condition.

### 3.3. Impact of Corrosion Temperature on the Tensile Strength Properties of Geomembrane Specimens

**Figure 4:**
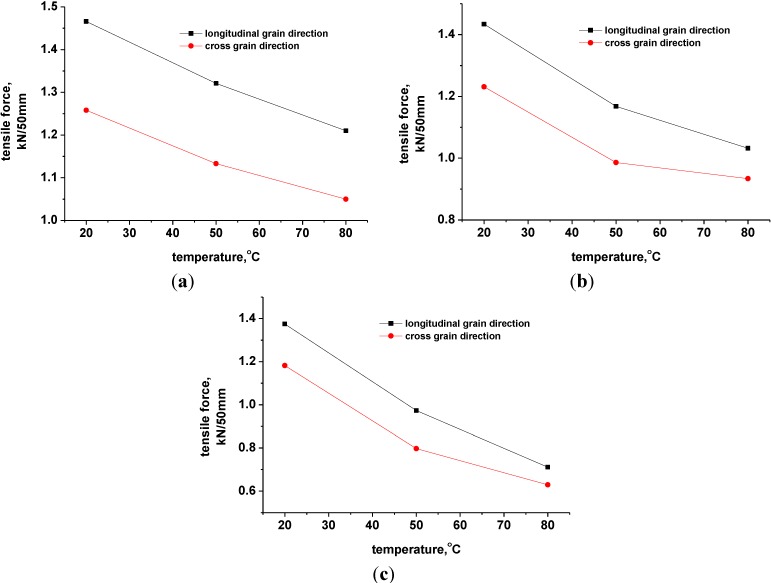
The tensile strength-temperature curves of geomembrane specimens. (**a**) 5 days; (**b**) 10 days; and (**c**) 20 days.

The corresponding tensile strengths of geomembrane specimens in the longitudinal grain direction were 1.466, 1.321 and 1.21 kN/50 mm, respectively, at corrosion temperature of 20, 50 and 80 °C and corrosion time of 5 days (Figure 4a). The corresponding tensile strengths of the geomembrane specimens in the cross grain direction were 1.258, 1.133 and 1.05 kN/50 mm. The tensile strengths in the longitudinal grain direction decreased by 0.27%, 10.14% and 17.69%, and the tensile strengths in the cross grain direction decreased by 0.94%, 10.79% and 17.32%. The corresponding tensile strengths of geomembrane specimens in the longitudinal grain direction were 1.375, 0.973 and 0.711 kN/50 mm, respectively, under corrosion temperatures of 20, 50 and 80 °C and corrosion time of 15 days (Figure 4c). The corresponding tensile strengths of the geomembrane specimens in the cross grain direction were 1.182, 0.797 and 0.629 kN/50 mm. The tensile strengths in the longitudinal grain direction decreased by 6.46%, 33.81% and 51.63%, and the tensile strengths in the cross grain direction decreased by 6.93%, 37.24% and 50.47%. As shown in Figure 4, the tensile strength of geomembrane gradually decreased with the corrosion temperature rising. As shown in Figure 4, the tensile strength of the geomembrane gradually decreased with the corrosion temperature rising. Furthermore, at the same time, the gap between cross grain direction and longitudinal grain direction also reduced. The aforementioned law was met under the corrosion time of 10 days condition.

The tensile strength was sensitive to the temperature. The maximum attenuation of the tensile strength was even over 50% when corrosion time was 15 days, which was attributed to the rising temperature causing the oxidative induction time to decrease more quickly [25] while enhancing the corrosion reaction between the leachate and the geomembrane polyethylene chains, which caused the polyethylene chains to break or decompose, resulting in the decrease of tensile strength. Thus, measures should be taken to control the temperature around the geomembrane and to prevent a high-temperature environment for a long time in the application.

### 3.4. The Puncture Strength Properties of Geomembrane Specimens Corroded with Leachate

**Figure 5:**
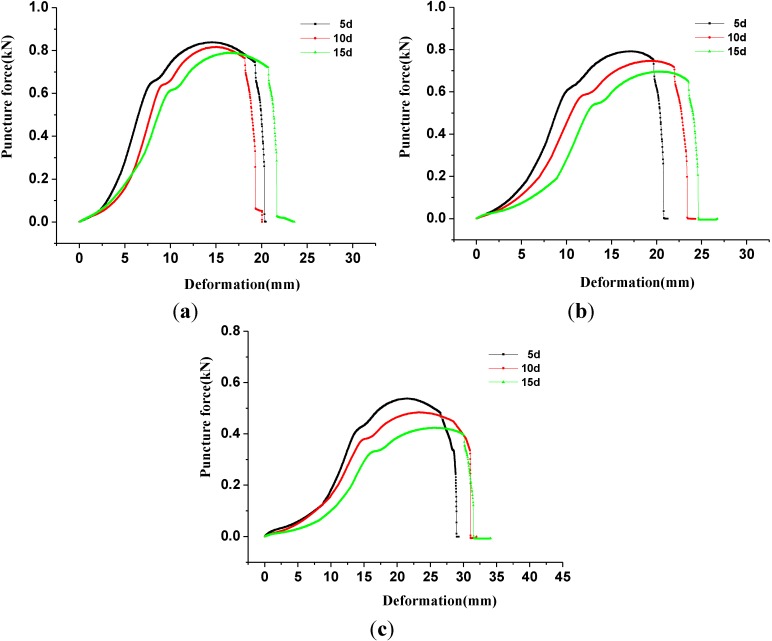
The puncture force-deformation curves of geomembrane specimens corroded with landfill leachate. (**a**) 20 °C; (**b**) 50 °C and (**c**) 80 °C.

Figure 5 illustrates the puncture force-deformation curves of geomembrane specimens corroded with leachate. At corrosion temperatures of 50 °C, the peaks of puncture force-deformation curves of specimens (aka the puncture strengths) were determined as 0.792, 0.746 and 0.695 kN after being corroded by leachate for 5, 10 and 15 days, respectively (Figure 5b). The deformations corresponding to the peak strength were 17.3, 18.8 and 20 mm, respectively. The puncture strengths decreased by 6.05%, 11.51% and 17.56% with the increase in corrosion time compared with the uncorroded geomembrane specimens, indicating the significant effect of corrosion time. Overall, the puncture force-deformation curves show the tendency of ductility with the increase in corrosion time, which indicated the reduction of puncture strength, but the deformation that corresponded to failure increased. Curves also exhibited a trend similar to that when the corrosion temperature was 20 °C (Figure 5a) and 80 °C (Figure 5b).

The corresponding puncture strengths were 0.817, 0.746 and 0.484kN, separately, at corrosion temperatures of 20, 50 and 80 °C with the same corrosion time of 10 days. The deformations corresponding to puncture strength were 15.1, 18.8 and 23.2 mm (Figure 5). By contrast, the puncture strengths decreased by 3.08%, 11.51% and 42.59%. The higher corrosion temperature was able to obviously affect the puncture-resistant behavior of HDPE geomembrane. Puncture strength was extremely sensitive to the corrosion temperature, thus the increase in corrosion temperature would induce a significant attenuation of the geomembrane puncture strength. The corresponding puncture strength, which was 0.423 kN (Figure 5c), was only half of that of uncorroded geomembrane specimens when the corrosion time was 15 days and the corrosion temperature reached 80 °C.

### 3.5. The Strength/Elongation/Deformation Variations of HDPE Geomembrane Specimens

The variations of vital properties of HDPE geomembrane regarding the tensile strength, residual strength, and elongation were compared with the uncorroded geomembrane specimens as listed in Table 4. It is shown that corrosion decreased tensile strength of geomembrane specimens. Residual strength increased slightly at low temperature such as 20 °C. However, once the temperature rose to 50 or 80 °C, the residual strengths of all the specimens exhibited the same behavior to that of the tensile strength, which indicated that residual strength increased with the increase in corrosion time and temperature. The elongation of the corroded geomembrane specimens increased compared with the uncorroded geomembrane specimens. The elongation variations increased initially and then decreased with higher temperature when corrosion time was constant. The temperature increase could destroy the long polyethylene chains in the geomembrane and the connection between chains to a certain extent, which would result in an ease to elongate. However, the elongation would gradually reduce when excessively high temperatures cause part of the polyethylene to decompose, which would induce different elongation variations at different corrosion times.

**Table 4:** The strength/elongation/deformation variations of geomembrane specimens corroded with leachate.

Project		Longitudinal grain direction			Cross grain direction		
		5 days	10 days	15 days	5 days	10 days	15 days
Tensile strength variations/kN	20 °C	0.004	0.036	0.095	0.012	0.039	0.088
	50 °C	0.149	0.302	0.497	0.137	0.284	0.473
	80 °C	0.260	0.438	0.759	0.220	0.336	0.641
Residual strength variations/kN	20 °C	−0.019	−0.001	0.009	−0.06	0.003	−0.002
	50 °C	0.037	0.148	0.243	0.055	0.184	0.324
	80 °C	0.166	0.254	0.530	0.124	0.180	0.486
Elongation variations/%	20 °C	0.6	0.4	0.6	0.1	0.9	1.3
	50 °C	0.9	1.3	1.6	1.4	2.1	2.5
	80 °C	1	0.7	0.3	1.3	0.9	0.5
**Project**		**Puncture strength variations/kN**			**Puncture deformation variations/mm**		
		5 days	10 days	15 days	5 days	10 days	15 days
Puncture test	20 °C	0.004	0.026	0.054	0.1	0.6	1.8
	50 °C	0.051	0.097	0.148	2.8	4.3	5.5
	80 °C	0.305	0.359	0.420	7.0	8.7	11.2

Note: to the tensile strength, elongation and puncture strength variations, decrease means positive, increase means positive for else.

As seen in Table 4, the value of various parameters increased with higher corrosion temperature or longer corrosion time, no matter whether accompanied with the reduction of puncture strength or with the growth of puncture deformation, which is consistent with the observation of the stress-strain curves that migrate upward and rightward (Figure 5).

## 4. Conclusions

(1) The tension and puncture failure of the HDPE geomembrane was progressive. Regardless of whether the geomembrane was in the longitudinal grain direction or in the cross grain direction, the tension force-deformation curves of geomembrane specimens rose quickly to the peak tensile strength at first, decreased gradually to minimum strength, then rose slowly again to approach the equilibrium, which corresponded to residual strength. Tensile strength in the longitudinal grain direction was obviously larger than that in the transverse direction. The geomembrane puncturing occurred shortly after the puncture force reached the puncture strength but not at the point of puncture strength.

(2) Under the 50 °C condition, the tensile strength in the longitudinal grain direction decreased by 10.79%, 20.54% and 33.81%; the tensile strength in the cross grain direction decreased by 6.05%, 22.36% and 37.24%; and the puncture strength decreased by 6.05%, 11.51% and 17.56% with the increase in corrosion time from 5 to 10 days. In both directions referring to the texture of geomembrane, the tensile strength of geomembrane decreased as corrosion time increased, while the variation in the cross grain direction was greater than that in the longitudinal grain direction. The puncture strength decreased with the increase in corrosion time, but the deformation of failure increased. These conclusions are also valid for the test cases at 20 and 80 °C.

(3) The tensile strength of the geomembrane decreased with corrosion temperature increases. The tensile strength of the geomembrane decreased by over 50% and the puncture strength was only half of the intact sample when the corrosion time was 15 days and the corrosion temperature was increased to 80 °C. Tensile strength and puncture strength proved to be extremely sensitive to temperature.

(4) In a word, the residual strength of the geomembrane decreased with the increase in corrosion time or corrosion temperature. The elongation of the corroded geomembrane was generally greater than that of the uncorroded one. The elongation variation increased and then decreased as the temperature increased. However, this differs from the impact of corrosion time. Both the reduction in puncture strength and the increase in puncture deformation had positive correlations with corrosion time or temperature. In conclusion, corrosion softened the geomembrane.
